# Supplementary material for: A structural biology community assessment of AlphaFold2 applications
Source: Nat Struct Mol Biol. 2022 Nov 7;29(11):1056–67. doi: 10.1038/s41594-022-00849-w (PMC9663297; doi:10.1038/s41594-022-00849-w)
Supplement: Supplementary file 1 — Supplementary Figures 1–11 and Supplementary Methods. [file 41594_2022_849_MOESM1_ESM.pdf]

nature structural & molecular biology

Article

https://doi.org/10.1038/s41594-022-00849-w

A structural biology community assessment of AlphaFold2 applications

In the format provided by the authors and unedited

Supplementary Information for : A structural biology community assessment of AlphaFold2 applications

Mehmet Akdel[1,*], Douglas E V Pires[2,*], Eduard Porta Pardo[3,4,*], Jürgen Jänes[5,*], Arthur O Zalevsky[6,*], Bálint Mészáros[7,*], Patrick Bryant[8,*], Lydia L. Good[9,*], Roman A Laskowski[5,*], Gabriele Pozzati[8], Aditi Shenoy[8], Wensi Zhu[8], Petras Kundrotas[8], Victoria Ruiz Serra[4], Carlos H M Rodrigues[2], Alistair S Dunham[5], David Burke[5], Neera Borkakoti[5], Sameer Velankar[5], Adam Frost[10], Jérôme Basquin[11], Kresten Lindorff-Larsen[9], Alex Bateman[5], Andrey V Kajava[12], Alfonso Valencia[4,#], Sergey Ovchinnikov[13,#], Janani Durairaj[14,#], David B Ascher[15,#], Janet M Thornton[5,#] Norman E Davey[16,#], Amelie Stein[9,#], Arne Elofsson[8,#], Tristan I Croll[17,#], Pedro Beltrao[5,18,#]

1 - Bioinformatics Group, Department of Plant Sciences, Wageningen University and Research, Netherlands
2 - School of Computing and Information Systems, University of Melbourne, Melbourne, Victoria, Australia
3 - Josep Carreras Leukaemia Research Institute (IJC),Badalona, Spain
4 - Barcelona Supercomputing Center (BSC)
5 - European Molecular Biology Laboratory, European Bioinformatics Institute (EMBL-EBI), Cambridge, UK.
6 - Shemyakin–Ovchinnikov Institute of Bioorganic Chemistry, Russian Academy of Sciences, 117997 Moscow, Russian Federation
7 - European Molecular Biology Laboratory, Heidelberg, Germany
8 - Dep of Biochemistry and Biophysics and Science for Life Laboratory, 17121 Solna, Sweden
9 - Linderstrøm-Lang Centre for Protein Science, Department of Biology, University of Copenhagen, DK-2200 Copenhagen N, Denmark
10 - Department of Biochemistry and Biophysics University of California, San Francisco
11 - Department of Structural Cell Biology, Max Planck Institute of Biochemistry, Am Klopferspitz 18, D-82152 Martinsried, Germany
12 - Université de Montpellier, Centre de Recherche en Biologie Cellulaire de Montpellier (CRBM) CNRS, UMR 5237, Montpellier, France
13 - Faculty of Arts and Sciences, Division of Science, Harvard University, Cambridge, MA 02138
14 - Biozentrum, University of Basel, Spitalstrasse 41, 4056 Basel, Switzerland
15 - School of Chemistry and Molecular Biology, University of Queensland, Brisbane, Queensland, Australia
16 - Institute of Cancer Research, London, UK
16 - Cambridge Institute for Medical Research, Department of Haematology, The University of Cambridge, Cambridge, UK
18 - Institute of Molecular Systems Biology, ETH Zürich, 8093 Zürich, Switzerland

* Authors contributed equally
# correspondence to: alfonso.valencia@bsc.es, so@fas.harvard.edu, janani.durairaj@gmail.com, d.ascher@uq.edu.au, thornton@ebi.ac.uk, norman.davey@icr.ac.uk, amelie.stein@bio.ku.dk, arne@bioinfo.se, tic20@cam.ac.uk, pbeltrao@ehtz.ch

Supplementary Figures

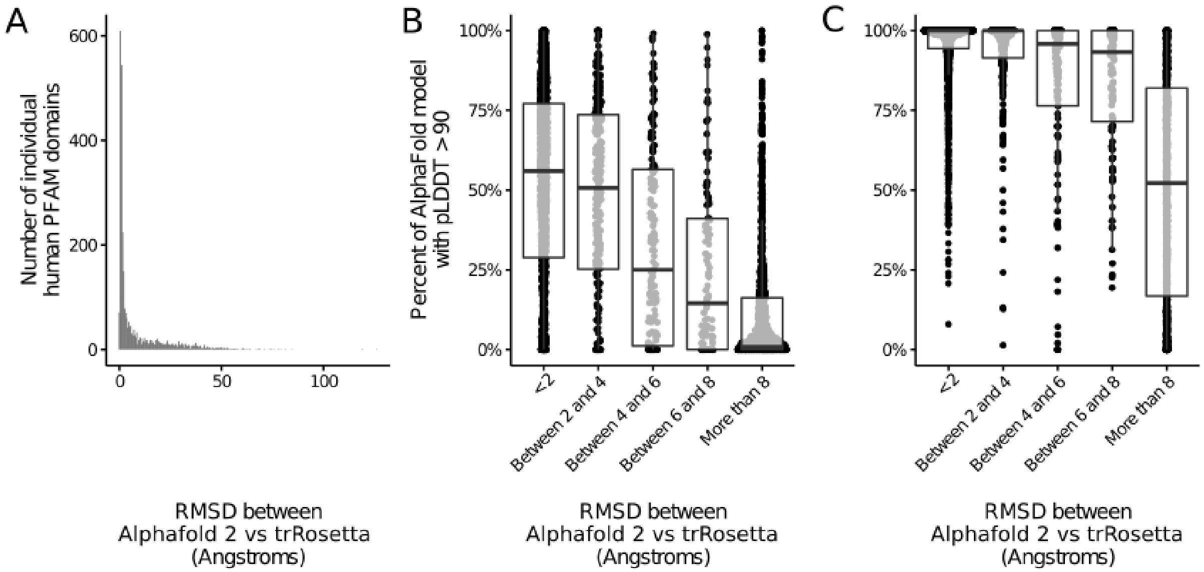

Supplementary Fig. 1 — Comparison between AF2 and trRosetta. A) Histogram showing how many AlphaFold models for human PFAM domains (y-axis) depending on their RMSD to the generic RoseTTAFold model for the same PFAM domain (x-axis). B) Boxplots showing, for each of the 3035 AlphaFold models of human PFAM domains (each dot) the percent of residues with a pLDDT above 90 (y-axis). AlphaFold models are grouped by their RMSD to the trRosetta generic PFAM model (x-axis). The bottom, middle line and top of the box correspond to the 25th, 50th and 75th percentile respectively. C) Same as B but for residues with pLDDT higher than 50. The bottom, middle line and top of the box correspond to the 25th, 50th and 75th percentile respectively.

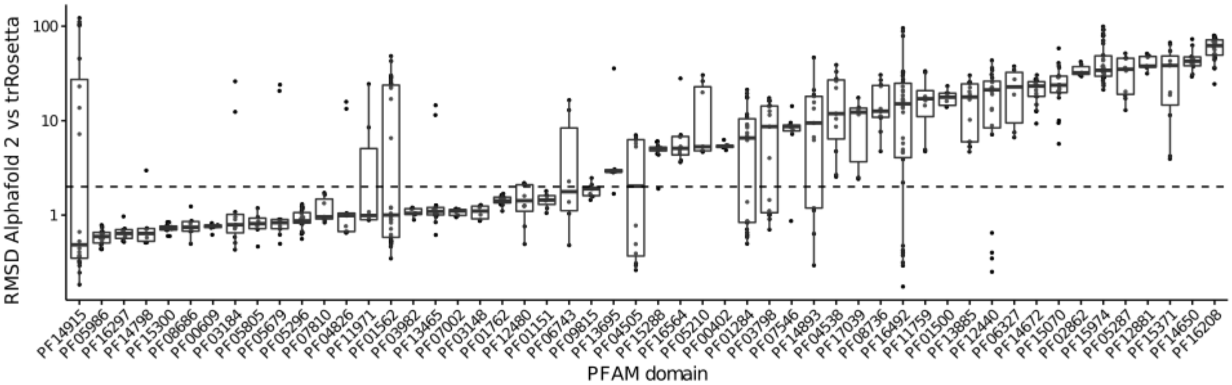

Supplementary Fig. 2 — Intradomain variability between the predicted models of each tool. Boxplots showing the RMSD between AlphaFold and trRosetta (y-axis, logarithmic) for different PFAM domain instances in the human proteome (dots). Instances are grouped by PFAM families (x-axis). The black dashed line indicates an RMSD of 2Å. The bottom, middle line and top of the box correspond to the 25th, 50th and 75th percentile respectively. The lines extend from the boxplot 1.5 * IQR (interquartile range).

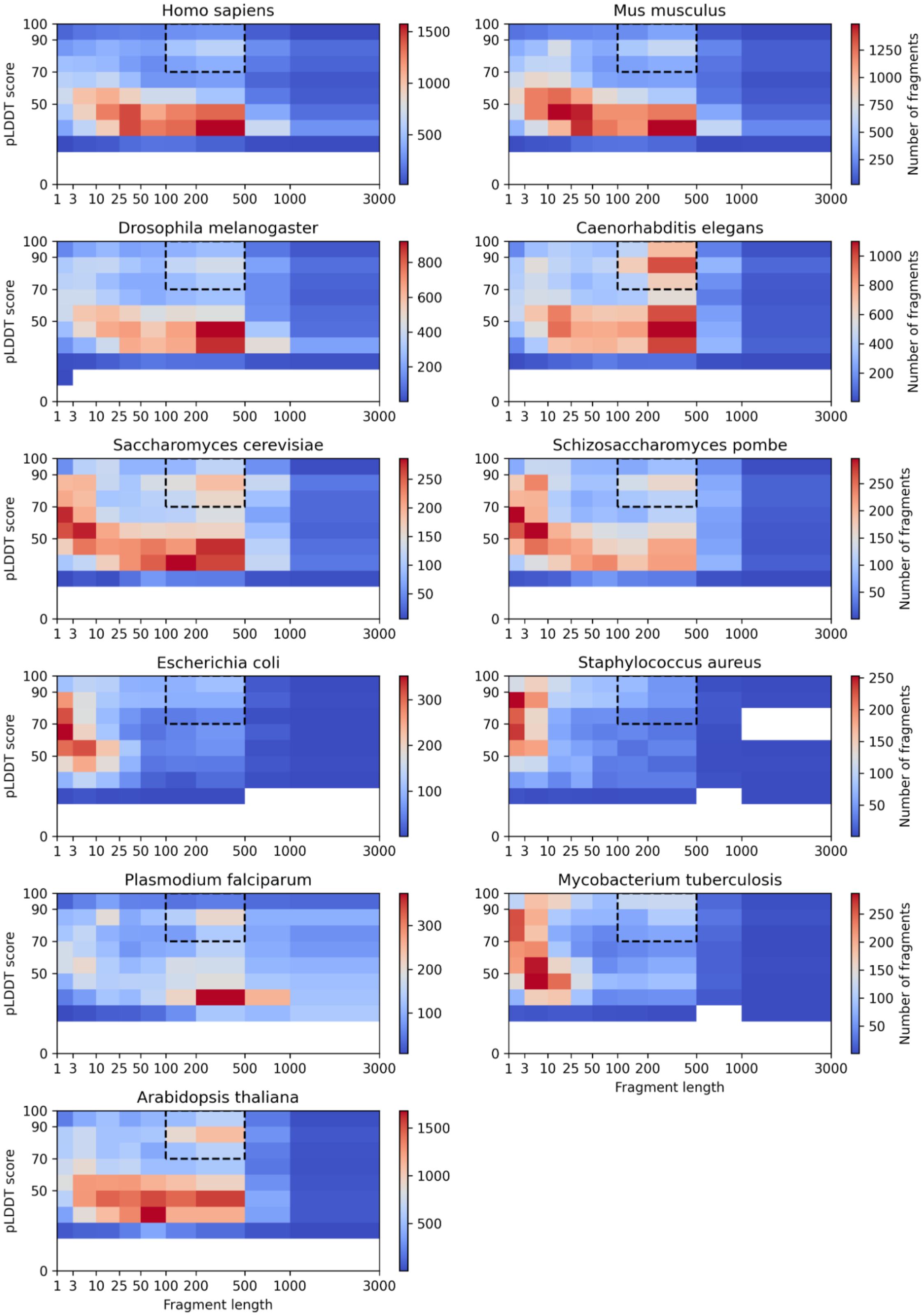
Homo sapiens
Mus musculus
Drosophila melanogaster
Caenorhabditis elegans
Saccharomyces cerevisiae
Schizosaccharomyces pombe
Escherichia coli
Staphylococcus aureus
Plasmodium falciparum
Mycobacterium tuberculosis
Arabidopsis thaliana
pLDDT score
0
50
70
90
100
1 3 10 25 50 100 200 500 1000 3000
Fragment length
Number of fragments

Supplementary Fig. 3 — Distribution of median pLDDT scores vs. Fragment length. Only fragments less than 3000 are shown. For most proteomes, we find some level of enrichment for high confidence fragments of length between 100 to 500 amino-acids (boxed areas).

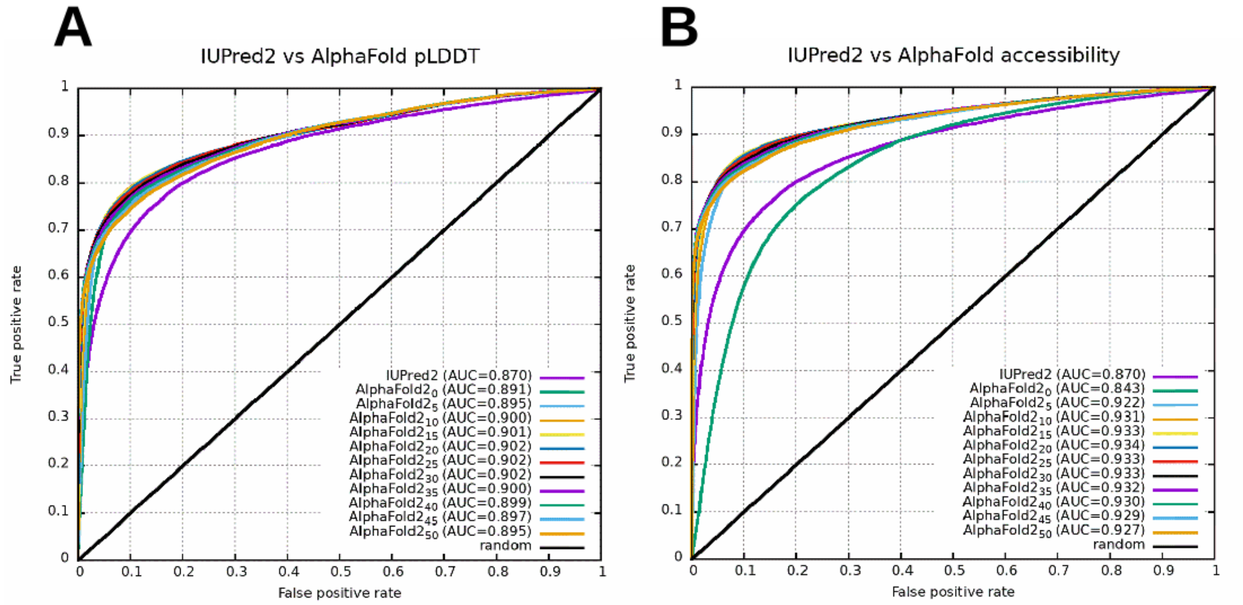

Supplementary Fig. 4 — Benchmarking AF2-derived measures against IUPred2. ROC curves comparing the pLDDT confidence scores. A) and calculated relative solvent accessible surface areas. B) of AF2 structure predictions to IUPred2. Indices in legends mark the window sizes used to smooth the values along the sequence. AUC = area under the curve, the overall measure of performance, where a perfect prediction is AUC=1.0 and a random prediction is AUC=0.5.

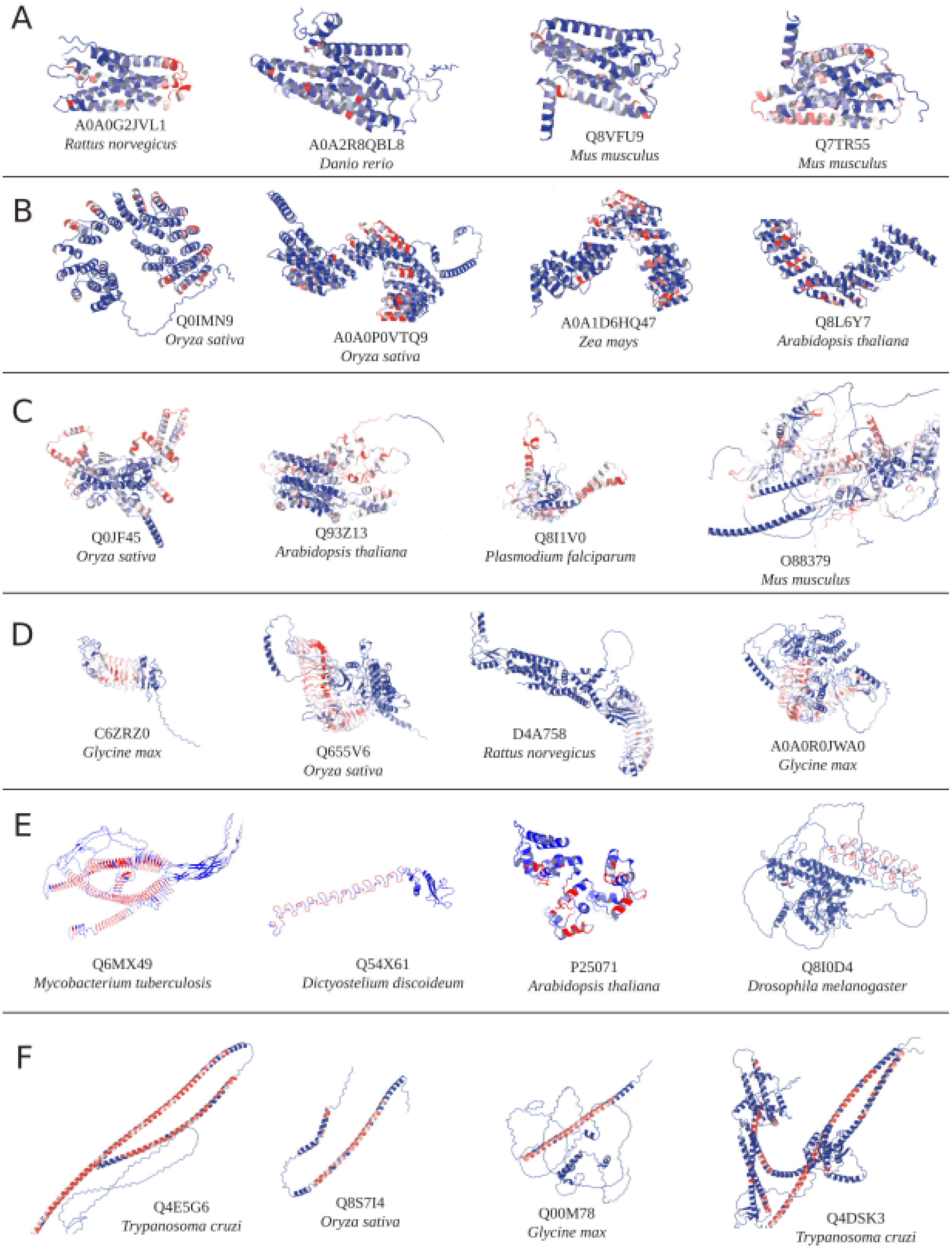

Supplementary Fig. 5 — Representative structures from topics representing proteins with specific combinations of structural elements that are poorly covered by experimental structures

deposited in PDB. Residues are colored according to their contribution to the topic under consideration - red residues have the highest contribution, while blue residues are specific to the example and not to the topic. A) GPCR olfactory receptors. B) Plant pentatricopeptide repeat proteins. C) ATP- and ion-binding proteins. D) Proteins with Leucine rich repeats. E) example structures from topic 188, including novel beta-solenoid structure predicted for a family of pentapeptide repeats in the mycobacterial PPE proteins F) Long α-helical constructs

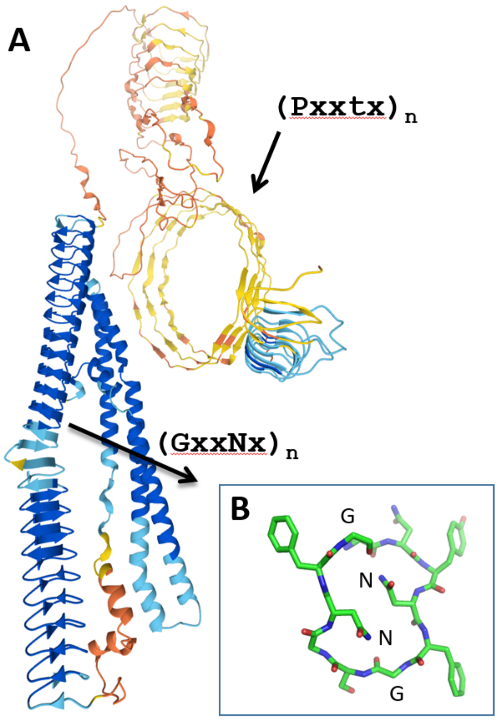

Supplementary Fig. 6 — Identification of a putative novel beta-solenoids structural element. A) A schematic representation of the AF2 structural model for PPE24_MYCTU protein. Arrows point to consensus sequences of the corresponding pentapeptide structures B) Axial projection of GXXNXGXXNX fragment representing the shortest possible coil of beta-solenoids.

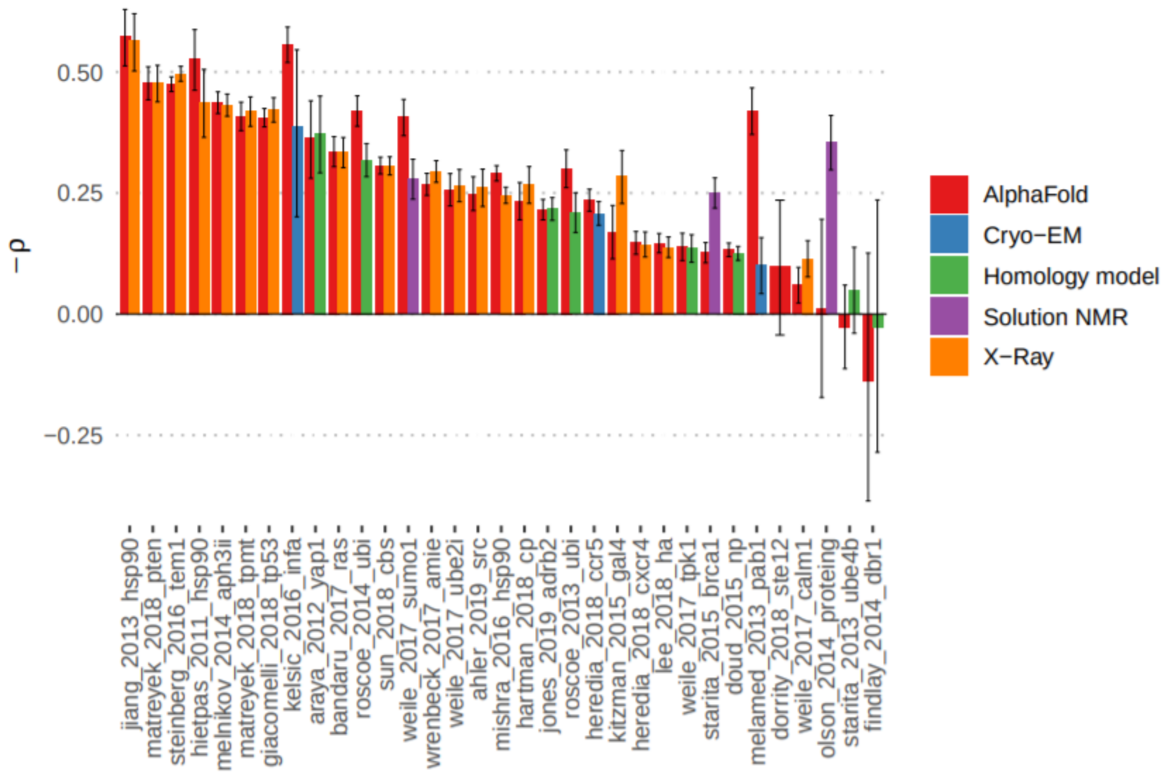

Supplementary Fig. 7 — Correlation between structure based prediction of destabilisation and measured impact on protein function by deep mutational scanning experiments. Relation between the predicted ΔΔG for mutations with measured experimental impact of the mutation from deep mutational scanning data (-1*pearson correlation). The predicted change in stability was done with FoldX using structures from AF2 or available experimental models. Data are presented as mean +/- the confidence intervals calculated via fisher's Z transform (R's cor.test function).

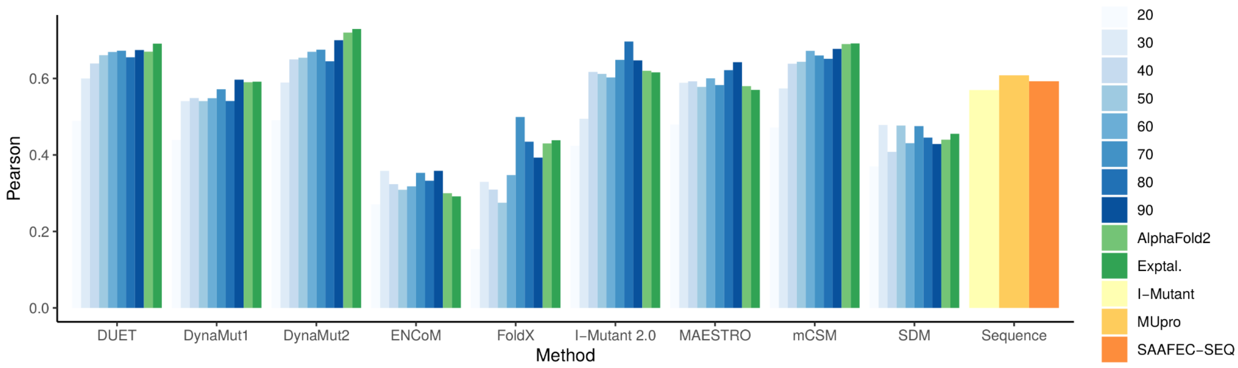

Fig S8 — Comparative performance of methods predicting stability changes upon mutation using AlphaFold2 and MODELLER models. The performance of nine well established structure-based methods is contrasted on a mutation benchmark data set when presented to either AlphaFold2 models (light green bars) or homology models (blue bars) derived from

templates of varying identity levels. Baseline performance using experimental structures (dark green bar) and of three sequence-based tools (yellow and orange bars) are also shown.

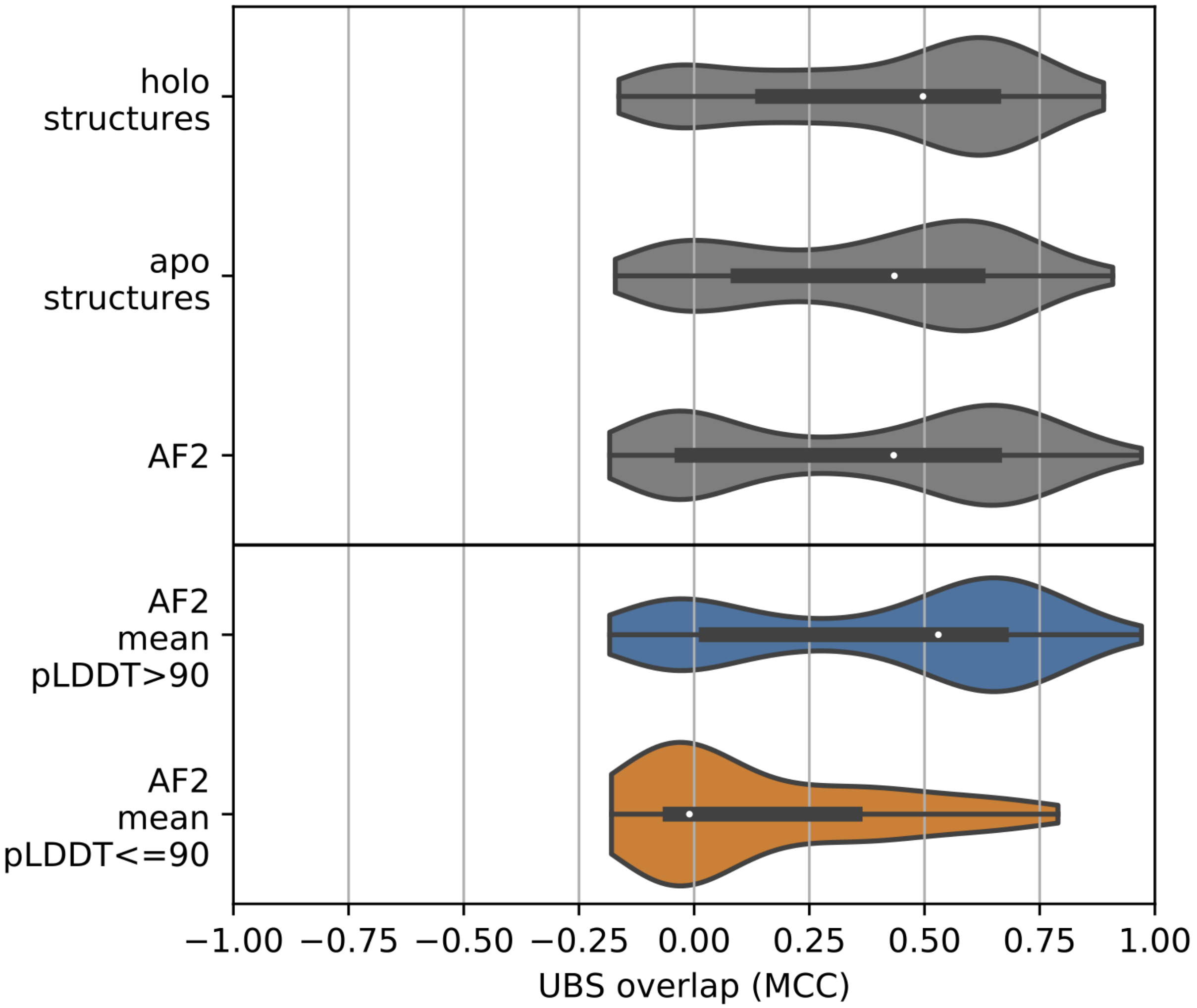

Supplementary Fig. 9 — Distribution of overlaps between known binding sites and top predicted pockets for holo, apo and AF2 structures, quantified using Matthew's Correlation Coefficients. The bottom, middle line and top of the box correspond to the 25th, 50th and 75th percentile respectively. The lines extend from the boxplot 1.5 * IQR (interquartile range).

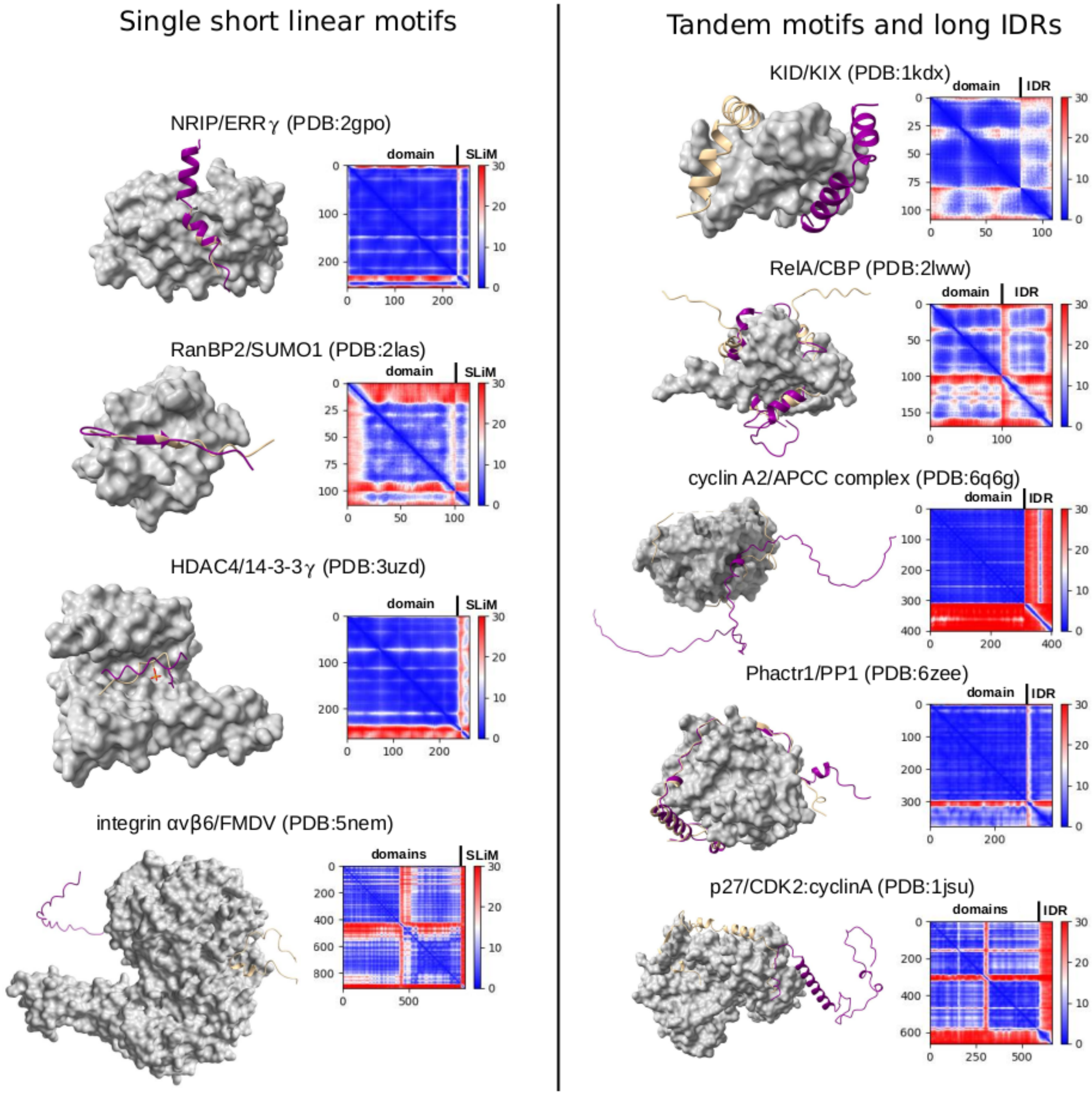

Supplementary Fig. 10 — AF2-predicted complex structures of single IDRs bound to ordered partners. Left - short linear motifs (SLiMs), from top to bottom: NRBOX motif in NRIP1 bound to ERR3, RanBP2 SIM bound to SUMO, HDAC4 14-3-3 phosphomotif bound to 14-3-3gamma, and the RGDLxxL motif of the FMDV viral protein bound to integrin alphavbeta6. Right — long IDRs with several binding regions, from top to bottom: KID region of CBP bound to the KIX domain, TAD of RelA bound to the TAZ domain of CBP, Cyclin-A2 bound to Cdc20, Phactr1 bound to PP1 and p27 bound to the CDK2:cyclinA complex.

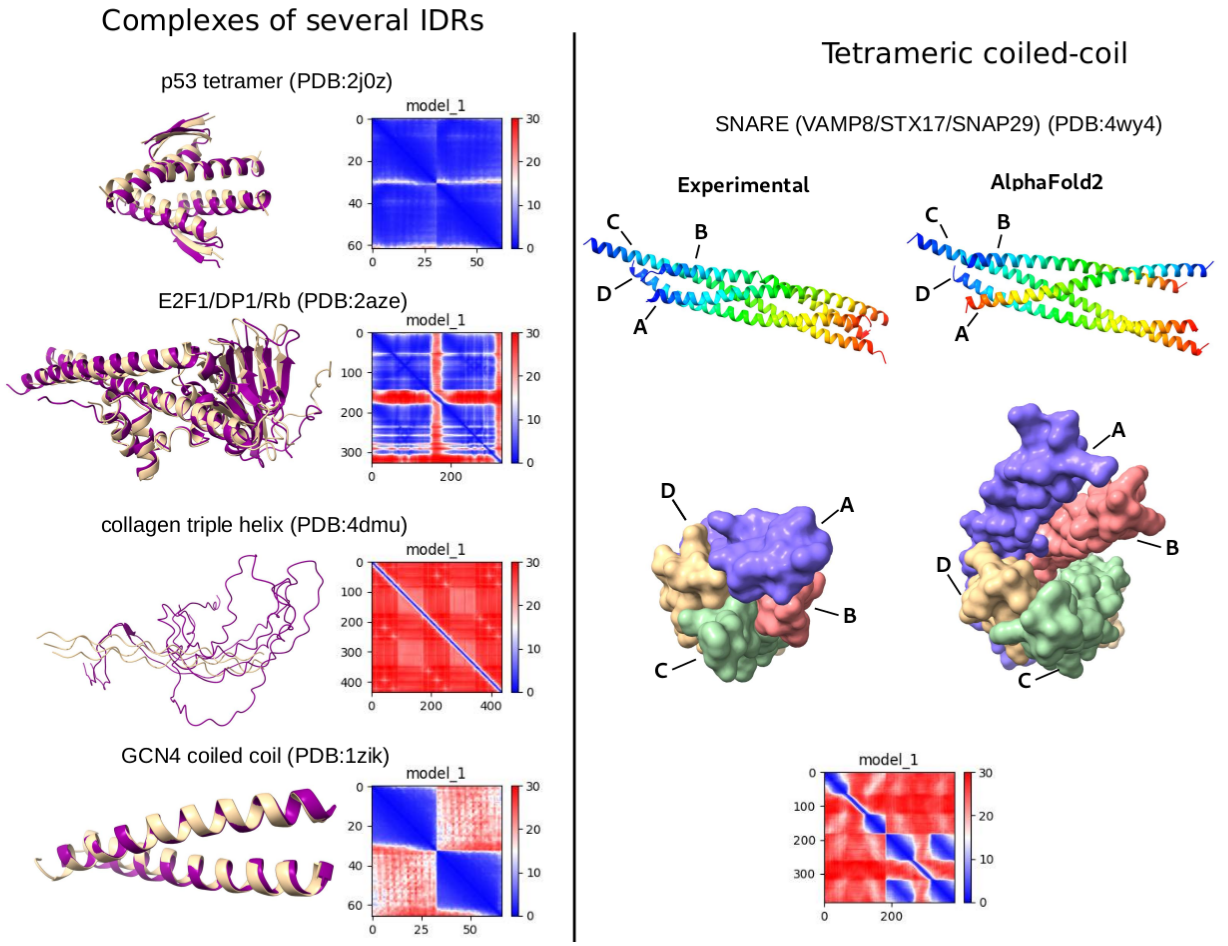

Supplementary Fig. 11 — AF2-predicted complex structures of complexes with multiple IDRs. Left — IDR-only complexes, from top to bottom: p53 tetramerization region, Rb:E2F1:DP1 heterotrimer, collagen triple helix and the GCN4 prototypic leucine zipper. Right: Autophagic SNARE core complex (Vamp8 / Syntaxin-17 / SNAP29).

Supplementary Methods

Prediction of variant effects with Rosetta

We began with a compilation of Deep Mutational Scanning (DMS) experiments[1] comprising 117,135 total mutations in 33 proteins. The AlphaFold structures for these proteins were pulled from the AlphaFold Protein Structure Database (https://alphafold.ebi.ac.uk), and the experimental structures were gathered from the and selected by balancing greatest available resolution and coverage -- both of the entire protein and specifically of the mutagenized region. We selected structures solved using X-ray crystallography when available. For proteins with no available experimental structures, we used the Swiss Model repository (Bienert et al 2017) and selected the homology model with the highest QMEANDisCo score. If the experimental structure contained multiple protein chains, we removed all other chains but the protein of interest. All

calculations were carried out using the Rosetta version with GitHub SHA `28f338acfb3bfd87048b38a04772486975dc83fa` from July 2, 2020.
We first relaxed the structures using the `relax` application and the following flags:

```
-fa_max_dis 9
-relax:constrain_relax_to_start_coords
-ignore_unrecognized_res
-missing_density_to_jump
-nstruct 1
-relax:coord_constrain_sidechains
-relax:cartesian
-beta
-score:weights beta_nov16_cart
-ex1
-ex2
-relax:min_type lbfgs_armijo_nonmonotone
-flip_HNQ
-no_optH false
```

Subsequently, we carried out saturation mutagenesis to calculate the change in protein folding stability (ΔΔG) for each single amino acid substitution using the Cartesian ΔΔG protocol and the `beta_nov16_cart` energy function with three iterations as previously described[2,3]. Flags for the ΔΔG calculations were:

```
-fa_max_dis 9.0
-ddg::dump_pdbs false
-ddg:iterations 3
-score:weights beta_nov16_cart
-missing_density_to_jump
-ddg:mut_only
-ddg:bbnbrs 1
-beta_cart
-ex1
-ex2
-ddg::legacy true
-optimize_proline true
```

Scores from the three iterations were averaged. Values of ΔΔG in Rosetta Energy Units were divided by 2.9 to bring them onto a scale corresponding to kcal/mol [2].

Supplementary References

1. Dunham, A. S. & Beltrao, P. Exploring amino acid functions in a deep mutational landscape. *Mol. Syst. Biol.* **17**, e10305 (2021).
2. Park, H. *et al.* Simultaneous Optimization of Biomolecular Energy Functions on Features from Small Molecules and Macromolecules. *J. Chem. Theory Comput.* **12**, 6201–6212 (2016).
3. Frenz, B. *et al.* Prediction of Protein Mutational Free Energy: Benchmark and Sampling Improvements Increase Classification Accuracy. *Front Bioeng Biotechnol* **8**, 558247 (2020).
